# Associations of Orthostatic Hypotension and Frailty With Dementia and Mortality in Older Adults: A Population-Based Cohort Study

**DOI:** 10.1093/gerona/glae010

**Published:** 2024-01-09

**Authors:** Xin Xia, Linus Jönsson, Clare Tazzeo, Chengxuan Qiu, Debora Rizzuto, Erika J Laukka, Giulia Grande, Laura Fratiglioni, Davide Liborio Vetrano

**Affiliations:** Aging Research Center, Department of Neurobiology, Care Sciences and Society (NVS), Karolinska Institutet–Stockholm University, Stockholm, Sweden; Section for Neurogeriatrics, Department of Neurobiology, Care Sciences and Society (NVS), Karolinska Institutet, Stockholm, Sweden; Aging Research Center, Department of Neurobiology, Care Sciences and Society (NVS), Karolinska Institutet–Stockholm University, Stockholm, Sweden; Aging Research Center, Department of Neurobiology, Care Sciences and Society (NVS), Karolinska Institutet–Stockholm University, Stockholm, Sweden; Aging Research Center, Department of Neurobiology, Care Sciences and Society (NVS), Karolinska Institutet–Stockholm University, Stockholm, Sweden; Stockholm Gerontology Research Center, Stockholm, Sweden; Aging Research Center, Department of Neurobiology, Care Sciences and Society (NVS), Karolinska Institutet–Stockholm University, Stockholm, Sweden; Stockholm Gerontology Research Center, Stockholm, Sweden; Aging Research Center, Department of Neurobiology, Care Sciences and Society (NVS), Karolinska Institutet–Stockholm University, Stockholm, Sweden; Aging Research Center, Department of Neurobiology, Care Sciences and Society (NVS), Karolinska Institutet–Stockholm University, Stockholm, Sweden; Stockholm Gerontology Research Center, Stockholm, Sweden; Aging Research Center, Department of Neurobiology, Care Sciences and Society (NVS), Karolinska Institutet–Stockholm University, Stockholm, Sweden; Stockholm Gerontology Research Center, Stockholm, Sweden; (Medical Sciences Section)

**Keywords:** Cognitive aging, Epidemiology, Public health

## Abstract

**Background:**

This study aimed to assess the associations of orthostatic hypotension (OH), in the presence or absence of frailty, with dementia and mortality in older adults.

**Methods:**

We conducted a 15-year population-based cohort study including 2 703 baseline dementia-free individuals from the Swedish National Study on Aging and Care in Kungsholmen. At baseline, OH was defined as a decline in systolic/diastolic blood pressure ≥20/10 mm Hg 1 minute after standing up from a supine position. Frailty status was defined following Fried’s frailty phenotype. Dementia was diagnosed following the Diagnostic and Statistical Manual of Mental Disorders-fourth edition criteria. Multistate flexible parametric survival models were used to estimate associations of OH and frailty with dementia and mortality.

**Results:**

Robust people with OH (adjusted hazard ratio [HR] = 2.28; 95% confidence interval [CI] = 1.47–3.54) and frail people without OH (HR = 1.98; 95% CI = 1.40–2.82) or with OH (HR = 2.73; 95% CI = 1.82–4.10) had a higher dementia risk than OH-free and robust people. Moreover, frail people, independently of the presence of OH, had higher mortality rate than OH-free and robust people. In individuals who developed dementia during the follow-up period, neither OH nor frailty was significantly associated with mortality.

**Conclusions:**

Older adults with OH, whether robust or frail, may have a higher dementia risk than those without OH. Older adults with OH, when having frailty, may have a higher mortality rate than those without OH. The concurrent assessments of OH and frailty may provide prognostic values in terms of dementia and mortality risk in older adults.

Orthostatic hypotension (OH) is an excessive decrease in blood pressure when standing up from a supine position (1). OH is prevalent in >20% of older adults and has been associated with a higher risk of several adverse health outcomes, such as dementia and mortality, possibly via repeated and chronic reductions in end-organ perfusion (1,2). However, most people with OH have preserved cerebral blood flow as a result of physiological adaptations and hemodynamic compensations (3), suggesting OH itself may not be enough to induce end-organ perfusion and subsequent damage.

Frailty is a common clinical syndrome in older adults characterized by decreased physiological reserve and increased vulnerability to stressors and often co-occurs with OH (4,5). Previous studies showed that people with frailty were more likely to have orthostatic intolerance, which is generally considered a result of insufficient cerebral perfusion (3,6). In addition, recent data showed that slow walking speed, an indicator of frailty, and multimorbidity, a risk factor for frailty, were both associated with impaired stabilization of cerebral oxygenation independently of arterial blood pressure response (7,8). It is possible that frailty adds to the effects of OH on end-organ perfusion and modifies the effects of OH on morbidity and mortality.

This study aimed to determine if OH can affect the risk of dementia and mortality independently of frailty. Specifically, we sought to investigate (i) if OH was associated with dementia and mortality risk when accompanied by or in the absence of frailty and (ii) if frailty could modify the effects of OH on the risk of dementia and mortality.

## Method

### Study Design and Study Population

The study is a population-based cohort study with a follow-up period from 2001–2004 (baseline) to 2016–2019, using data from the Swedish National Study on Aging and Care in Kungsholmen (SNAC-K). In brief, the SNAC-K is an ongoing project consisting of residents in the central area of Stockholm, Kungsholmen, randomly selected from 11 age cohorts: 60, 66, 72, 78, 81, 84, 87, 90, 93, 96, and 99+ years. At baseline, 3 363 (73.3%) of 4 590 eligible and invited people underwent the first examination. Participants aged 60–72 years were then followed every 6 years, and participants aged ≥78 years were followed every 3 years. At each study visit, participants underwent structured interviews, clinical examinations, and cognitive testing by trained nurses, physicians, and psychologists. Diseases and symptoms were recorded using the International Classification of Diseases (ICD) codes; medication use was recorded according to the Anatomical Therapeutic Chemical (ATC) Classification System (9). The Swedish National Patient Register and the Swedish Cause of Death Register were obtained and linked to SNAC-K. All parts of SNAC-K, including the linkage to the 2 Swedish registers, were approved by the Regional Ethical Review Board in Stockholm, and written informed consent was obtained from each participant or their proxy if the participant was cognitively impaired.

In the present study, participants with dementia at baseline (*n* = 240), missing dementia diagnosis at baseline (*n* = 10), or with Parkinson’s disease at baseline or the third-year follow-up study visit (*n* = 43) were excluded. Of the 3 070 baseline participants free of dementia and Parkinson’s disease, 367 participants withdrew (refused participation, lost contact, or moved) before the first follow-up study visit, leaving 2 703 (88.0%) participants in the final analytical sample.

### Assessments of OH and Frailty

Blood pressure was measured from the left arm with a sphygmomanometer by trained physicians in a quiet room with a constant temperature. Supine blood pressure was measured after a 5-minute rest. Then, the participants stood up, and blood pressure in the standing position was measured after 1 minute. OH was defined as a decrease in systolic blood pressure (SBP) ≥20 mm Hg or diastolic blood pressure (DBP) ≥10 mm Hg 1 minute after standing up from the supine position.

Frailty was defined according to Fried’s frailty phenotype, which considers 5 conditions: unintentional weight loss, weak grip strength, self-reported exhaustion, slow walking speed, and low physical activity level (4). People without any of the conditions were defined as robust; people having 1–2 of the 5 conditions were defined as prefrail; people having 3–5 of the 5 conditions were defined as frail (4). Prefrailty is a stage that is different from robustness and predisposes to frailty (4). The operationalization of the frailty phenotype has been previously described elsewhere (10). Briefly, weight loss was ascertained through interviews by nurses, and unintentional weight loss was defined as losing at least 1 kg in the last 3 months. The grip strengths of both hands were assessed with an electronic dynamometer under the instruction of trained nurses, and the stronger grip strength value was used. Weak grip strength was defined as the lowest 20% sex-and-body mass index-adjusted grip strength of the participants. For participants without grip strength assessment, weak grip strength was defined as incapable of opening jars with lids. Self-reported exhaustion was defined as feeling fatigued in the last 3 months. Walking speed was measured through a 6-m or 2.4-m walk test, and slow walking speed was defined as the slowest 20% sex-and-height-adjusted walking speed of the participants. Physical activity was assessed through a self-administrated questionnaire, and low physical activity level was defined as exercising 3 times per month or less. We chose frailty phenotype over frailty index, another commonly used frailty instrument, because cognitive impairment is an important component in the frailty index but is a main outcome of interest in our study. In addition, the frailty phenotype focuses on physical pathophysiological aspects, whereas the frailty index involves multiple domains, including psychosocial aspects, which are not the focus of the study. Prefrailty and frailty were later combined due to the limited number of participants with frailty, which would result in small numbers of dementia and death cases in participants with both OH and frailty. In addition, prefrailty also confers excessive risk of morbidity (11). Prefrailty and frailty will later be referred to as frail for brevity.

### Ascertainment of Dementia and Death

Dementia was diagnosed according to the Diagnostic and Statistical Manual of Mental Disorders-fourth edition criteria following a 3-step procedure. In brief, the examining physician made the first diagnosis based on interviews of health histories (clinical histories and medication use), general physical examinations, neurological examinations, cognitive tests (the Mini-Mental State Examination, the clock drawing test, the Digit Span Forward and Backward tests, items regarding memory, executive functioning, problem-solving, orientation, and interpretation of proverbs, and a story comprehension test assessing frontal lobe function administered by nurses), and interviews of activities of daily living. Another physician blinded to the first diagnosis made a second diagnosis by reviewing the records of interviews, clinical examinations, and cognitive tests. In the case of discrepancies between the first and second diagnoses, a neurologist external to the data collection made the final diagnosis. For participants who died between consecutive study visits without a prior dementia diagnosis, a physician made a dementia diagnosis by reviewing medical charts. In addition, the Swedish Cause of Death Register was linked to SNAC-K to identify dementia cases in those participants (ICD-10 codes: F00, F01, F02, F03, and G30).

Vital status and the date of death were ascertained through the Swedish Cause of Death Register and by the SNAC-K staff.

### Measurements of Covariates

Educational level and smoking status were ascertained through a questionnaire administrated by nurses. Educational level was divided into elementary, high school, and university levels. Smoking status was divided into current smoking (yes/no). Weight and height were measured in light clothes without shoes, and body mass index was calculated and divided into underweight, normal weight, overweight, and obese. Blood pressure in the sitting position was measured twice, each after a 5-minute rest, from the left arm with a sphygmomanometer, and the mean of the 2 blood pressure measurements was used. Hypertension was defined as SBP/DBP ≥140/90 mm Hg or taking antihypertensive medications (ATC codes: C02, C03, C04, C07, C08, and C09). Peripheral blood samples were collected, and diabetes was defined as self-reported diabetes, having a record of diabetes diagnosis in the Swedish National Patient Register, having glycated hemoglobin A1c (HbA1c) ≥6.5%, or using antidiabetic medications (ATC code: A10). Atrial fibrillation, ischemic heart disease, heart failure, and cerebrovascular disease were ascertained by physicians in the SNAC-K through interviews of medical histories, reviewing medical charts, clinical examinations, lab parameters, and reviewing currently used medications and identified by records of diagnoses in the Swedish National Patient Register (9).

### Statistical Analysis

Participants were divided into 4 groups according to their OH and frailty status: OH-free and robust, OH-free and frail, with OH and robust, and with OH and frail. The baseline characteristics of participants in the 4 groups were described and compared by multinomial logistic regressions with all variables of the characteristics included as the independent variables and OH–frailty status as the dependent variable. We also tested the association between OH and frailty using logistic regressions. Characteristics of the SNAC-K participants included in the present study and participants who withdrew from the study were compared using logistic regressions.

We used multistate flexible parametric survival models with follow-up time as the timescale to examine the associations of OH and frailty with transitions from no dementia to dementia and death. Robust people without OH were the reference group. Hazard ratios (HRs) and 95% confidence intervals (CIs) are reported. Participants were followed from baseline to death, withdrawal, or the end of the study (December 2019), whichever came first. The models were adjusted for age, sex, education, smoking status, body mass index categories, hypertension, diabetes, atrial fibrillation, ischemic heart disease, heart failure, and cerebrovascular disease. We tested the potential modifying effects of frailty on the associations of OH with dementia and mortality on the multiplicative scale by including OH, frailty, and their interaction term in fully adjusted survival models and on the additive scales by calculating relative excess risk due to interaction (RERI). In addition, we conducted stratified analyses by frailty status to evaluate the associations of OH with dementia and mortality in the strata of frailty status. Finally, we predicted cumulative incidences and transition probabilities of dementia and death by OH and frailty status after fitting the fully adjusted flexible parametric survival models. The cumulative incidences can be interpreted as the probability of having experienced an event by specified time points. The transition probabilities can be interpreted as the probability of being in a state at specific time points given being in a state at a specific time point previously.

We conducted the following sensitivity analyses: (i) separating prefrailty from frailty in the flexible parametric survival models with the exception of when the survival outcome was mortality in incident dementia cases due to the small number of events; (ii) defining OH as a decrease in SBP/DBP ≥30/15 mm Hg after standing up from the supine position in people with supine hypertension (supine SBP/DBP ≥150/90 mm Hg) to test the robustness of our results to changing OH to an alternative definition (12); (iii) adjusting for categories of sitting blood pressure levels and use of antihypertensives; (iv) additionally adjusting for the use of antidepressants (ATC code: N06A) to evaluate the possible confounding effects of this factor; (v) excluding individuals that developed dementia or died within 3 years after the baseline OH and frailty assessments to test the impact of reverse causation. We did not do this for the analysis of the transition from dementia to death, as many people with dementia died within 3 years after the dementia diagnosis.

Missing values in OH, frailty, and covariates were imputed using the method of multiple imputation by chained equations. Details of the statistical analyses are provided in Supplementary Material. Data were prepared and analyzed using R software version 4.2.2 (R Foundation for Statistical Computing, Vienna, Austria) and Stata Statistical Software: Release 17.0 (StataCorp, College Station, TX).

## Results

The average age of the study population was 73.7 years (standard deviation [*SD*], 10.8), and 63.3% were females (Table 1). People who withdrew from the study after the baseline study visit were younger, had lower educational levels, and were more likely to smoke currently but otherwise comparable to people who attended follow-up study examinations (Supplementary Table 1). At baseline, 825 (30.5%) people were OH-free and robust, 969 (35.8%) were OH-free and frail, 233 (8.6%) had OH and were robust, and 328 (12.1%) had OH and were frail (Table 1). OH and frailty were not significantly associated in adjusted logistic regression analyses (Supplementary Table 2).

**Table 1.** Baseline Characteristics of the Study Population by OH and Frailty Status

Characteristics	OH-Free and Robust (*n* = 825)	OH-Free and Frail (*n* = 969)	With OH and Robust (*n* = 233)	With OH and Frail (*n* = 328)	Overall (*n* = 2 703)
Age (years), mean (*SD*)	68.4 (7.9)	74.5 (10.5)^†^^,^*	71.0 (8.6)^†^^,^*	77.4 (10.7)^†^^,^*	73.7 (10.8)
Sex: female, *n* (%)	502 (60.8)	592 (61.1)	153 (65.7)	218 (66.5)	1 710 (63.3)
Education, *n* (%)					
Elementary (reference)	72 (8.7)	168 (17.3)	26 (11.2)	68 (20.7)	421 (15.6)
High school	369 (44.7)	499 (51.5)	131 (56.2)	163 (49.7)	1 350 (49.9)
University	384 (46.5)	302 (31.2)^‡^^,^*	76 (32.6)	97 (29.6)	925 (34.2)
Body mass index categories, *n* (%)					
Underweight (<18.5 kg/m^2^)	4 (0.5)	23 (2.4)^†^^,^*	3 (1.3)	16 (4.9)^†^^,^*	72 (2.7)
Normal weight (18.5–24.9 kg/m^2^, reference)	353 (42.8)	380 (39.2)	117 (50.2)	147 (44.8)	1 142 (42.2)
Overweight (25–29.9 kg/m^2^)	379 (45.9)	372 (38.4)	85 (36.5)^‡^^,^*	117 (35.7)	1 022 (37.8)
Obese (≥30 kg/m^2^)	87 (10.5)	161 (16.6)^†^^,^*	24 (10.3)	31 (9.5)	332 (12.3)
Current smoking, *n* (%)	112 (13.6)	148 (15.3)^†^^,^*	27 (11.6)	51 (15.5)^†^^,^*	385 (14.2)
Hypertension, *n* (%)	574 (69.6)	754 (77.8)	158 (67.8)	247 (75.3)	2 008 (74.3)
SBP/DBP <140/90 mm Hg and not using antihypertensives	244 (29.6)	204 (21.1)	71 (30.5)	76 (23.2)	660 (24.4)
SBP/DBP <140/90 mm Hg and using antihypertensives	62 (7.5)	110 (11.4)	11 (4.7)	60 (18.3)^†^^,^*	320 (11.8)
SBP/DBP ≥140/90 mm Hg and not using antihypertensives	333 (40.4)	322 (33.2)	66 (28.3)^‡^^,^*	93 (28.4)^‡^^,^*	889 (32.9)
SBP/DBP ≥140/90 mm Hg and using antihypertensives	176 (21.3)	320 (33.0)^†^^,^*	79 (33.9)	94 (28.7)	784 (29.0)
Diabetes, *n* (%)	48 (5.8)	94 (9.7)	18 (7.7)	41 (12.5)^†^^,^*	248 (9.2)
Atrial fibrillation, *n* (%)	37 (4.5)	87 (9.0)	19 (8.2)	35 (10.7)	249 (9.2)
Ischemic heart disease, *n* (%)	63 (7.6)	160 (16.5)^†^^,^*	22 (9.4)	70 (21.3)^†^^,^*	401 (14.8)
Heart failure, *n* (%)	18 (2.2)	95 (9.8)^†^^,^*	7 (3.0)	53 (16.2)^†^^,^*	264 (9.8)
Cerebrovascular disease, *n* (%)	31 (3.8)	56 (5.8)	5 (2.1)	39 (11.9)^†^^,^*	185 (6.8)

*Notes*: DBP = diastolic blood pressure; OH = orthostatic hypotension; SBP = systolic blood pressure; *SD* = standard deviation. The numbers of missing data are 180 for OH, 218 for frailty status, 7 for education, 135 for body mass index categories, 26 for smoking, 35 for hypertension, and 50 for categories of sitting blood pressure levels and use of antihypertensives.

^†^More likely to be in the category.

^‡^Less likely to be in the category.

*p < .05.

**Figure 1.**
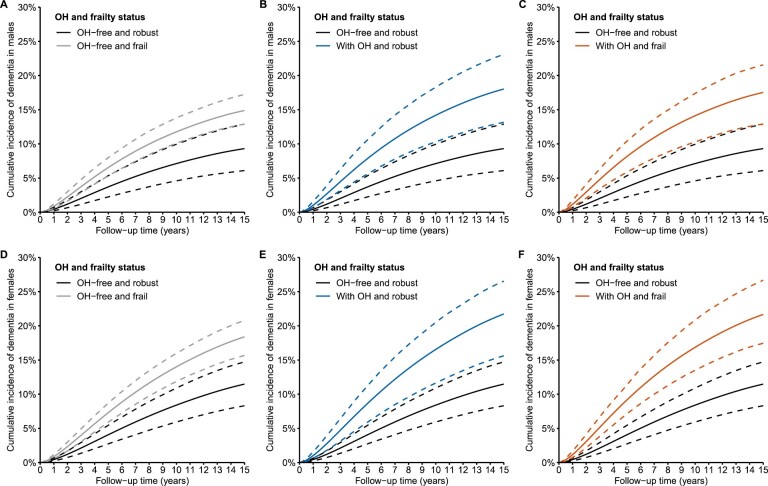
Standardized cumulative incidence of dementia by orthostatic hypotension (OH) and frailty status. (A–C) Standardized cumulative incidence of dementia in males; (D–F) standardized cumulative incidence of dementia in females. Dashed lines are 95% confidence intervals.

During an average of 8.9 (*SD*, 4.6) years of follow-up, 390 (14.4%) people developed dementia. People with OH or frailty had a higher hazard of dementia than OH-free and robust people (Table 2). When stratifying by frailty status, the association between OH and dementia was more evident in robust people (HR = 2.28; 95% CI = 1.47–3.54) than in frail people (HR = 1.38; 95% CI = 0.97–1.78), although there were no significant modifying effects of frailty on either the multiplicative or additive scale (Table 2). This result should be interpreted cautiously, as the baseline risk of dementia was lower in robust people than in frail people (Figure 1), which may be the reason why the association between OH and dementia appeared to be stronger in robust people.

**Table 2.** Associations of OH and Frailty With Dementia and Death

	OH-Free		With OH	
	No. Outcome	HR (95% CI)	No. Outcome	HR (95% CI)
Dementia as the outcome^†^				
Robust	41	1.0 (reference)	33	2.28 (1.47–3.54)*
Frail	153	1.98 (1.40–2.82)*	69	2.73 (1.82–4.10)*
Death without dementia as the outcome^‡^				
Robust	166	1.0 (reference)	53	1.13 (0.84–1.52)
Frail	357	1.28 (1.05–1.54)*	144	1.56 (1.25–1.96)*
Transition from dementia to death as the outcome^§^				
Robust	24	1.0 (reference)	23	1.49 (0.84–2.63)
Frail	107	1.30 (0.85–2.00)	51	1.14 (0.71–1.85)

*Notes*: CI = confidence interval; HR = hazard ratio; OH = orthostatic hypotension; RERI = relative excess risk due to interaction. Results are from multistate flexible parametric survival models with OH-free and robust people as the reference group. HRs (95% CIs) were adjusted for age, sex, education, smoking status, body mass index categories, hypertension, diabetes, atrial fibrillation, ischemic heart disease, heart failure, and cerebrovascular disease.

^†^HR of the interaction term between OH and frailty = 0.64 (0.38 to 1.06), *p* value = .085. Measure of effect modification on additive scale (RERI) = −0.54 (−1.60 to 0.53), *p* value = .321.

^‡^HR of the interaction term between OH and frailty = 1.13 (0.79 to 1.60), *p* value = .512. Measure of effect modification on additive scale (RERI) = 0.15 (−0.28 to 0.59), *p* value = .482.

^§^HR of the interaction term between OH and frailty = 0.60 (0.31 to 1.14), *p* value = .118. Measure of effect modification on additive scale (RERI) = −0.66 (−1.64 to 0.32), *p* value = .189.

*p < .05.

During the follow-up period, 910 (33.7%) died without dementia. Robust people with OH did not have a higher hazard of death without dementia than OH-free and robust people (Table 2). In contrast, OH-free and frail people and frail people with OH had a higher hazard of death without dementia (Table 2). When stratifying by frailty status, OH was marginally associated with death without dementia in frail individuals (HR = 1.23, 95% CI = 1.00–1.46) but not in robust people (HR = 1.13; 95% CI = 0.84–1.52). There were no evident modifying effects of frailty on the association between OH and risk of death without dementia on either multiplicative or additive scales (Table 2).

Among people who developed dementia, 38 people withdrew from the study or reached the end of the study. In the remaining 352 dementia cases, 278 died during the follow-up period. OH seemed to be associated with higher mortality rate in robust people who later developed dementia, but the association was not statistically significant (Table 2). When stratifying by frailty status, OH seemed to be associated with a lower mortality rate in frail people (HR = 0.88; 95% CI = 0.59–1.17). Notably, the proportion of frailty in frail people without OH who later developed dementia was larger than in frail people with OH who later developed dementia (Supplementary Table 3).

The standardized 15-year cumulative incidence of dementia in people with either OH or frailty was higher than in OH-free and robust people, and the differences were slightly more evident in females than in males (Figure 1 and Supplementary Table 4). The standardized cumulative incidences of death without dementia in people with OH or frailty were not evidently higher than in OH-free and robust people (Supplementary Figure 1 and Supplementary Table 4).

**Figure 2.**
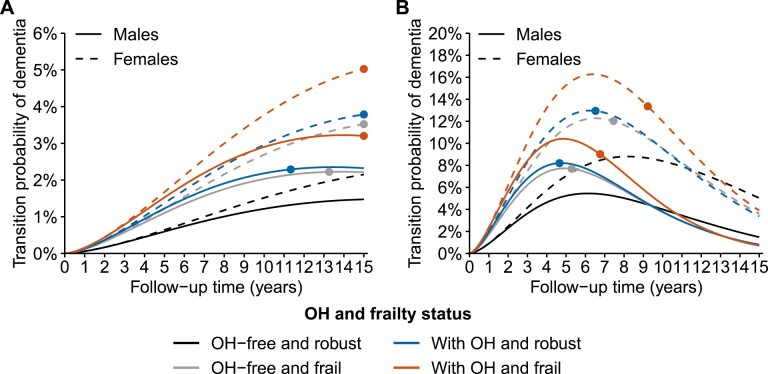
Transition probability of dementia in males and females by orthostatic hypotension (OH) and frailty status. (A) Transition probability of dementia for people aged 65 years; (B) transition probability of dementia for people aged 85 years. Solid circles indicate when the differences in transition probabilities compared with the OH-free and robust group stop being significant. Predictions at age 65 years are made for people with a university educational level, not smoking currently, having normal weight, having hypertension, and without diabetes, atrial fibrillation, ischemic heart disease, heart failure, and cerebrovascular disease. Predictions at age 85 years are made for people with the same characteristics except for the educational level being high school level. The values of the covariates were chosen based on their mean or most frequent values.

The transition probabilities of dementia for people aged 65 years with either OH or frailty were higher than robust people without OH, and the differences were larger and spanned over a longer period in females than males (Figure 2A). The transition probabilities of dementia in people aged 85 years had similar patterns (Figure 2B). In people aged 65 years, the transition probabilities of death were higher in frail people but not in robust people with OH than robust people without OH (Supplementary Figure 2A). Similarly, for people aged 85 years, the transition probabilities of death were higher in frail people than in OH-free and robust people. In addition, robust people with OH seemed to have a higher transition probability of death than OH-free and robust people after approximately 4 years (Supplementary Figure 2B).

When separating prefrailty from frailty, the association between OH and dementia was stronger in robust people than in prefrail or frail people (Supplementary Table 5). However, these results should be interpreted with caution, as the number of events in frail people with OH was very small, which may lead to sparse data bias. In contrast, the association between OH and death without dementia seemed to be more evident in frail people than in robust and prefrail people (Supplementary Table 5). In the sensitivity analyses that took supine hypertension into account when defining OH, the OH–dementia association in frail people (HR = 1.54; 95% CI = 1.06–2.02) and the modifying effects of frailty on the association between OH and mortality risk in people who developed dementia on the multiplicative scale became statistically significant (Supplementary Table 6). The sensitivity analyses when (i) adjusting for categories of sitting blood pressure levels and use of antihypertensives, (ii) additionally adjusting for the use of antidepressants, and (iii) when excluding dementia or death cases occurring within the first 3 years of the follow-up period did not produce significantly different results from the main analyses; however, the modifying effects of frailty on the association between OH and dementia on the multiplicative scale became statistically significant (Supplementary Tables 7–9).

## Discussion

This population-based cohort study in adults aged ≥60 years found that (i) OH was associated with elevated dementia risk in robust as well as in frail older adults and (ii) OH was associated with risk of death without dementia only in the presence of frailty. Our study indicates that OH predicts dementia risk even in the absence of frailty. In contrast, OH predicts increased mortality rate only in frail people.

### Comparisons With Previous Studies

The associations of OH and frailty with dementia observed in our study are in line with previous studies, which suggested that both OH and frailty, even prefrailty, were associated with elevated dementia risk (11,13–27). However, the potential modifying effects of frailty on the OH–dementia association have not been explored before. Our study adds to the current literature that OH, both with and without frailty, is associated with elevated dementia risk.

Although the relationship between frailty and mortality is well-established, the association between OH and mortality is less clear (4,28–30). The discrepancies in the OH–mortality associations in previous studies can be partly explained by differences in confounding factors controlled for in those studies (28–30). Our study controlled for demographic factors, vascular risk factors, and cardiovascular comorbidities and found that OH was only associated with shorter survival without dementia when accompanied by frailty. Future studies that include a larger number of frail participants are needed to confirm our results.

Few studies have investigated the clinical relevance of OH in people with dementia and reported inconsistent findings (31–33). One study found that symptomatic OH predicted fall risk in dementia patients (31). Another study showed that impaired orthostatic blood pressure recovery was associated with an accelerated mortality rate in dementia patients (32). In contrast, 1 study did not find an association between OH and survival times in dementia patients (33). On the other hand, a few studies on the effects of frailty on health outcomes in dementia patients have associated frailty with higher risk of hospitalization and mortality (34–36). Our study showed that OH was associated with mortality in individuals who developed dementia, though the association was not statistically significant. In addition, frailty seemed to attenuate the association between OH and mortality in individuals who developed dementia. The latter finding should be interpreted with caution because we combined prefrailty and frailty, and the proportion of frailty in frail dementia patients with OH was lower than in frail dementia patients without OH.

### Potential Mechanisms

OH and frailty have similar pathophysiological basis considering that OH can result from the incapacity of multiple organ systems (eg, cardiovascular system, nervous system) to deal with the stressor of blood pressure fluctuations due to postural changes, whereas frailty reflects accumulating deficits of organ systems (1,4). The associations of OH and frailty with dementia are complex and may involve multiple mechanisms. Shared risk factors such as cardiovascular risk factors, cardiovascular diseases, and underlying brain pathologies can partly account for the associations of OH and frailty with dementia (1,37,38). Our study showed that OH and frailty were strongly associated with dementia, even when adjusting for cardiovascular risk factors and diseases and when limiting dementia cases to those that developed dementia at least 3 years after the OH and frailty assessment. This suggests that shared risk factors may not fully explain the associations of OH and frailty with dementia.

Alternatively, OH and frailty may affect dementia development. OH can lead to reduced perfusion and subsequent ischemic injury in the heart and brain and has been associated with elevated risk of cardiovascular diseases, which may then increase dementia risk (3,39–46). Frailty reflects reduced physiological reserve and increased vulnerability to stressors and is considered a marker of biological aging (4,47). People with frailty have been shown to have impaired stabilization of cerebral oxygenation during postural change, which can be independent of the arterial blood pressure response (7,8). In addition, previous research suggested that frailty may lower the threshold of brain pathologies needed to trigger the clinical presentation of dementia (23,48). It is plausible that frailty may amplify the effects of OH on dementia. However, our study did not find additive modifying effects of frailty on the OH–dementia association. Future studies are warranted to confirm our results given that in our study, the number of people with frailty was small, leading to a small number of incident dementia cases in frail people with OH.

Several plausible pathways can account for the associations between OH and mortality. OH has been associated with increased risk of cardiovascular diseases, which can increase mortality risk (44,45). OH was also related to a higher risk of falls, which confers elevated risk of disability and mortality (49). A recent study showed that combining orthostatic SBP decrease with frailty improved the prediction of fall risk (50). This is in line with the finding of our study that OH was associated with increased mortality risk only in frail people who were dementia-free at baseline. Contrarily, our study found that, in people who developed dementia during the follow-up period, frailty attenuated the association of OH with mortality. A plausible explanation for the lack of association between combined OH and frailty and mortality in dementia patients is that frail dementia patients with OH may be less mobile and thus at a lower risk of falling and lower mortality risk. However, this finding should be examined in studies that can separate prefrailty from frailty when cross-classifying OH and frailty status.

### Study Limitations

The main limitation of the study is that due to the small number of people with frailty, prefrailty and frailty were combined. It is possible that frailty has stronger modifying effects on the associations of OH with dementia and mortality than prefrailty. Therefore, future studies that separate prefrailty and frailty are needed to further investigate these associations. Second, the number of incident dementia cases in each OH–frailty group in our study is small, and the analyses of the associations of OH and frailty with mortality in dementia patients may be underpowered. Third, due to the small numbers of people with OH when cross-classifying OH and frailty, we were unable to consider subtypes of OH such as asymptomatic and symptomatic OH, although asymptomatic and symptomatic OH may confer different risks of dementia and mortality. Fourth, our study only measured OH after 1 minute of standing and could miss people with delayed OH, which may have stronger effects on the study outcome. Fifth, our study did not investigate time-varying OH and frailty due to lack of frequent assessments and considering the increased susceptibility of time-varying analyses to difference biases (eg, reverse causation, biases due to missing data). Six, there exists residual confounding from imperfectly measured confounding factors (eg, self-reported smoking status) and unmeasured confounding factors (eg, neuropathological burden). In addition, we did not have the information to ascertain the history of hypertension, whether hypertension in our study population was controlled or uncontrolled, and if it was affected by white coat hypertension. This may have led to insufficient control of confounding bias and overestimating the associations of OH and frailty with dementia and mortality. Lastly, our study population has a relatively higher socioeconomic status than other regions in Sweden, and the study findings may not generalize to other populations.

## Conclusion

Our long-term population-based cohort study suggests that older adults with OH, whether robust or frail, may have a higher dementia risk than those without OH. OH may not affect mortality rate in the absence of frailty in older adults.

Our study suggests that OH may carry prognostic values for dementia independently of frailty status in older adults. Managing OH in both robust and frail people may help reduce the risk of dementia. In addition, OH is associated with a higher mortality rate when accompanied by frailty. The concurrent assessments of OH and frailty may aid in the risk prediction of dementia and mortality in older adults. Future studies that separate frailty from prefrailty are warranted to confirm our results.

## Supplementary Material

Supplementary data are available at *The Journals of Gerontology, Series A: Biological Sciences and Medical Sciences* online.

glae010_suppl_Supplementary_Material

## Data Availability

The anonymized data of the SNAC-K are accessible when the steering group of the SNAC-K approves the request (snac-k.se).
