# Supplementary material for: Associations of Orthostatic Hypotension and Frailty With Dementia and Mortality in Older Adults: A Population-Based Cohort Study
Source: J Gerontol A Biol Sci Med Sci. 2024 Jan 9;79(4):glae010. doi: 10.1093/gerona/glae010 (PMC10919881; doi:10.1093/gerona/glae010)
Supplement: glae010_suppl_Supplementary_Material [file glae010_suppl_supplementary_material.pdf]

Contents

Supplementary Methods 2

Table S1. Baseline characteristics of SNAC-K participants included in and withdrew from the study 5

Table S2. Associations of prefrailty and frailty with OH 6

Table S3. Numbers of people with prefrailty and frailty in analytical samples for different transitions 7

Table S4. Standardised cumulative incidences of dementia and death without dementia by OH and frailty status at year 15 8

Table S5. Associations of OH and frailty with dementia and death, separating prefrailty from frailty 9

Table S6. Associations of OH and frailty with dementia and death, considering supine hypertension in OH 10

Table S7. Associations of OH and frailty with dementia and death, adjusted for categories of sitting blood pressure levels and use of antihypertensives 11

Table S8. Associations of OH and frailty with dementia and death, additionally adjusted for the use of antidepressants 12

Table S9. Associations of OH and frailty with dementia and death, excluding dementia and death cases in the first three years of the follow-up period 13

Figure S1. Standardised cumulative incidence of death without dementia by OH and frailty status. 14

Figure S2. Transition probability of death in males and females by OH and frailty status. 15

Supplementary Methods

Multistate flexible parametric survival models

Multistate flexible parametric survival models were fitted with a user-written Stata command "Stmerlin" (1). In our study, the three-state multistate flexible parametric survival models can be seen as three separated flexible parametric survival models (**Figure 1**): (1) a flexible parametric survival model with dementia as the outcome of interest in people originally free of dementia; (2) a flexible parametric survival model with death without dementia as the outcome of interest in people originally free of dementia; (3) a flexible parametric survival model with death as the outcome of interest in people who developed dementia during the follow-up period. The follow-up time of each flexible parametric survival model for each transition is illustrated in **Figure 2**. The degrees of freedom used for modelling baseline cumulative hazards in each of the flexible parametric survival models were chosen based on the Bayesian information criterion and visually comparing predicted cumulative hazards with Nelson–Aalen estimators for the cumulative hazards.

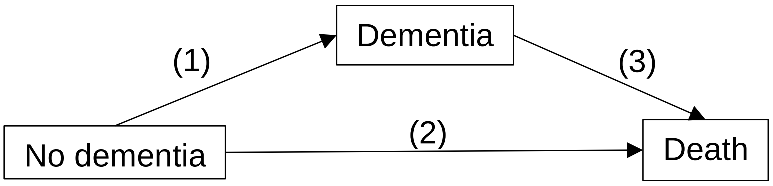

Figure 1. The three-state multistate models

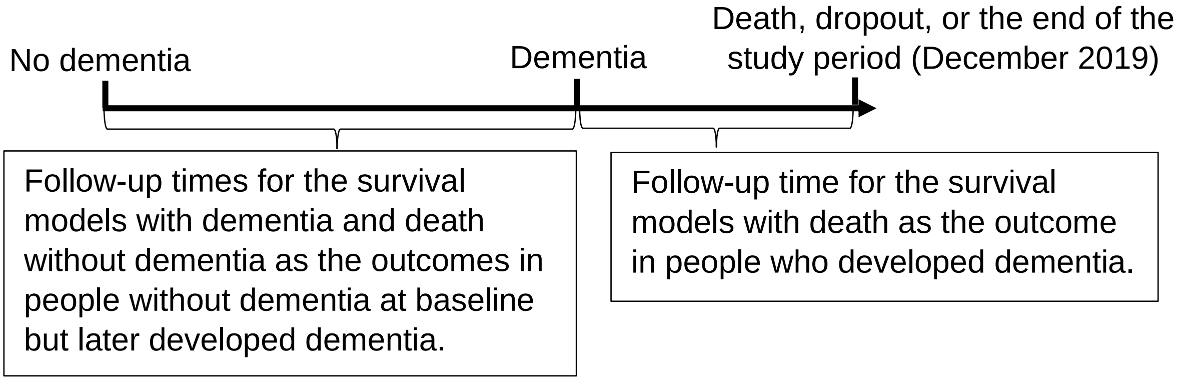

Figure 2. Illustration of follow-up times in the three-state multistate models. Note: the follow-up times in survival models with dementia and death without dementia as the outcomes for people who did not develop dementia is the period between the start of the study and time of death, dropout, or the end of the study period (December 2019), whichever came first.

Prediction of cumulative incidence

After fitting flexible parametric survival models with dementia and death without dementia as the outcomes of interest, i.e., transitions 1 and 2 in **Figure 1**, cumulative incidences of dementia and death without dementia were predicted using baseline hazards and hazard ratios estimated from the two sets of survival models (2). We used a user-written Stata command "standsurv" to predict standardised (marginal) cumulative incidences by orthostatic hypotension (OH) and frailty status, which facilitate comparisons between groups by forcing the distributions of covariates to be the same across the groups (2). This is done by predicting a cumulative incidence for each individual in the study with values of covariates set to their

observed values (except for the variable that defines the group of interest) and then averaging the individual cumulative incidences (2). To be able to use “standsurv”, we used another command, “stpm2” instead of “stmerlin”, which generated the same baseline hazards and hazard ratios as “stmerlin”. We changed the statistical command for fitting flexible parametric survival models only when predicting cumulative incidences and only because “standsurv” does not support post-estimation from “stmerlin”.

Prediction of transition probability

After fitting flexible parametric survival models for all transitions, i.e., transitions 1, 2, and 3 in **Figure 1**, transition probabilities of dementia and death can be estimated by integrating the baseline hazards and hazard ratios from all three transitions (1). The transition probability can be interpreted as the probability of being in a state at a specific time point. We used “predictms” command to predict conditional transition probabilities of dementia and death after fitting the multistate flexible parametric survival models with “stmerlin” (1). We predicted conditional transition probabilities of dementia and death at ages 65 and 85 years for males and females separately. When predicting transition probabilities by OH and frailty status at age 65 years, we specified the following values for covariates: university level, not smoking currently, normal weight, having hypertension, and without diabetes, atrial fibrillation, ischemic heart disease, heart failure, and cerebrovascular disease. When predicting transition probabilities at age 85 years, we used the same covariate values, except for education level, which was specified as a high school level. We chose conditional predictions over standardised predictions considering the computational burden of standardised predictions. We chose predictions at ages 65 and 85 years based on the mean baseline ages of the younger cohorts and the older cohorts in the SNAC-K. We specified the values of other covariates based on their most frequent value.

Imputation for missing data

We dealt with missing data in OH, frailty, and covariates with multiple imputation by chained equations (3). For each flexible parametric survival model, we performed multiple imputation separately and included all variables that were later included in the analysis models in the imputation models. For instance, for the fully adjusted analysis for the association of OH and frailty with dementia when considering the interaction between OH and frailty, we included the following variables in the imputation model: OH, frailty, interaction term between OH and frailty, age, sex, education, smoking status, body mass index categories, hypertension, diabetes, atrial fibrillation, ischemic heart disease, heart failure, cerebrovascular disease, Nelson-Aalen estimate of the cumulative hazard function of dementia, and an indicator of whether an individual developed dementia or not. Categorical variables were imputed with ordinal logistic regressions, and binary variables were imputed with logistic regressions. We generated 20 imputed datasets for each flexible parametric survival model. We used Rubin’s rules to pool hazard ratios from multistate models and standardised cumulative incidences from “standsurv” and their confidence intervals. We used a bootstrap followed by multiple imputation approach to pool point estimates and confidence intervals of transition probabilities predicted from the imputed datasets (4).

References

1. Crowther MJ, Lambert PC. Parametric multistate survival models: Flexible modelling allowing transition-specific distributions with application to estimating clinically useful measures of effect differences. Stat Med. 2017;36(29):4719-42.
2. Mozumder SI, Rutherford MJ, Lambert PC. Estimating restricted mean survival time and expected life-years lost in the presence of competing risks within flexible parametric survival models. BMC Med Res Methodol. 2021;21(1):52.
3. White IR, Royston P, Wood AM. Multiple imputation using chained equations: Issues and guidance for practice. Stat Med. 2011;30(4):377-99.
4. Bartlett JW, Hughes RA. Bootstrap inference for multiple imputation under uncongeniality and misspecification. Stat Methods Med Res. 2020;29(12):3533-46.

Table S1. Baseline characteristics of SNAC-K participants included in and withdrew from the study

Characteristics	Included (n = 2703)	Withdrawal (n = 367)
Age (years), mean (SD)	73.7 (10.8)	71.9 (10.0)[a]
Sex-female, n (%)	1710 (63.3)	234 (63.8)
Education, n (%)		
Elementary (reference)	421 (15.6)	65 (17.7)
High school	1350 (49.9)	174 (47.4)[b]
University	925 (34.2)	121 (33.0)[b]
Body mass index categories, n (%)		
Underweight (<18.5 kg/m^2)	72 (2.7)	6 (1.6)
Normal weight (18.5-24.9 kg/m^2, reference)	1142 (42.2)	146 (39.8)
Overweight (25–29.9 kg/m^2)	1022 (37.8)	147 (40.1)
Obese (≥30 kg/m^2)	332 (12.3)	47 (12.8)
Current smoking, n (%)	385 (14.2)	64 (17.4)[a]
Orthostatic hypotension, n (%)	614 (22.7)	66 (18.0)
Frailty status, n (%)		
Robust (reference)	1075 (39.8)	150 (40.9)
Prefrail	1134 (42.0)	153 (41.7)
Frail	276 (10.2)	34 (9.3)
Hypertension, n (%)	2008 (74.3)	278 (75.7)
SBP/DBP<140/90 mm Hg and not using antihypertensives	660 (24.4)	83 (22.6)
SBP/DBP<140/90 mm Hg and using antihypertensives	320 (11.8)	33 (9.0)
SBP/DBP≥140/90 mm Hg and not using antihypertensives	889 (32.9)	143 (39.0)[a]
SBP/DBP≥140/90 mm Hg and using antihypertensives	784 (29.0)	98 (26.7)
Diabetes, n (%)	248 (9.2)	36 (9.8)
Atrial fibrillation, n (%)	249 (9.2)	28 (7.6)
Ischaemic heart disease, n (%)	401 (14.8)	46 (12.5)
Heart failure, n (%)	264 (9.8)	21 (5.7)
Cerebrovascular disease, n (%)	185 (6.8)	24 (6.5)

The numbers of missing data are 14 for education, 156 for body mass index categories, 35 for current smoking, 207 for orthostatic hypotension, 248 for frailty status, 41 for hypertension, and 60 for categories of blood pressure and use of antihypertensives.
[a]P-value <0.05.
[b]P-value <0.01.
The P-values are from logistic regressions with all variables included in the logistic regressions, and the reference group was the participants included in the study.
DBP = diastolic blood pressure; SBP = systolic blood pressure.

Table S2. Associations of prefrailty and frailty with OH

	No. OH	Odds ratio (95% confidence interval)	
		**Model 1**	**Model 2**
**Prefrailty/frailty vs. robustness**			
**Robustness (n = 1075)**	233	1.0 (reference)	1.0 (reference)
**Prefrailty/frailty (n = 1410)**	328	1.02 (0.82-1.25)	0.98 (0.79-1.22)
**Prefrailty and frailty vs. robustness**			
**Robustness (n = 1075)**	233	1.0 (reference)	1.0 (reference)
**Prefrailty (n = 1134)**	255	0.97 (0.78-1.20)	0.95 (0.77-1.18)
**Frailty (n = 276)**	73	1.24 (0.88-1.73)	1.12 (0.79-1.58)

Results are odds ratios from logistic regressions with OH as the dependent variable and frailty status as the independent variable.
Model 1 adjusted for age, sex, and education; model 2 additionally adjusted for smoking status, body mass index categories, hypertension, diabetes, atrial fibrillation, ischaemic heart disease, heart failure, and cerebrovascular disease.
OH = orthostatic hypotension.

Table S3. Numbers of people with prefrailty and frailty in analytical samples for different transitions

	Robust	Prefrail	Frail
**Transition from no dementia to dementia or from no dementia to death**			
**OH-free and robust (n = 825)**	825	-	-
**OH-free and frail (n = 969)**	-	825 (85.1%)	144 (14.9%)
**With OH and robust (n = 233)**	233	-	-
**With OH and frail (n = 328)**	-	255 (77.7%)	73 (22.3%)
**Transition from dementia to death**			
**OH-free and robust (n = 56)**	56	-	-
**OH-free and frail (n = 160)**	-	118 (73.8%)	42 (26.3%)
**With OH and robust (n = 35)**	35	-	-
**With OH and frail (n = 73)**	-	57 (78.1%)	16 (21.9%)

OH = orthostatic hypotension.

Table S4. Standardised cumulative incidences of dementia and death without dementia by OH and frailty status at year 15

	**Males**	**Females**
**Cumulative incidences of dementia**		
**OH-free and robust**	9% (6%-13%)	11% (8%-15%)
**OH-free and frail**	15% (13%-17%)	18% (16%-21%)
**With OH and robust**	18% (13%-23%)	22% (16%-27%)
**With OH and frail**	18% (13%-22%)	22% (17%-27%)
**Cumulative incidences of death without dementia**		
**OH-free and robust**	49% (45%-53%)	37% (34%-41%)
**OH-free and frail**	50% (46%-53%)	38% (34%-41%)
**With OH and robust**	46% (39%-53%)	34% (29%-40%)
**With OH and frail**	51% (47%-58%)	39% (35%-45%)

OH = orthostatic hypotension.

Table S5. Associations of OH and frailty with dementia and death, separating prefrailty from frailty

	OH-free		With OH		HR (95% CI) for OH across frailty status
	No. outcome	HR (95% CI)	No. outcome	HR (95% CI)	
**Dementia as the outcome**					
**Robust**	41	1.0 (reference)	33	2.33 (1.49-3.62)[a]	2.33 (1.49-3.62)[a]
**Prefrail**	111	1.82 (1.28-2.58)[a]	53	2.72 (1.82-4.08)[a]	1.50 (1.00-2.00)[b]
**Frail**	42	3.30 (2.08-5.24)[a]	16	2.93 (1.62-5.30)[a]	0.90 (0.37-1.42)
**Death without dementia as the outcome**					
**Robust**	166	1.0 (reference)	53	1.16 (0.87-1.56)	1.16 (0.87-1.56)
**Prefrail**	279	1.22 (1.00-1.48)[b]	94	1.43 (1.09-1.87)[a]	1.17 (0.90-1.45)
**Frail**	78	1.64 (1.23-2.18)[a]	50	2.41 (1.68-3.44)[a]	1.47 (0.94-2.00)

Results are from multistate flexible parametric survival models with OH-free and robust people as the reference group. HRs (95% CIs) were adjusted for age, sex, education, smoking status, body mass index categories, hypertension, diabetes, atrial fibrillation, ischaemic heart disease, heart failure, and cerebrovascular disease.

[a]P-value <0.01.

[b]P-value <0.05.

CI = confidence interval; HR = hazard ratio; OH = orthostatic hypotension.

Table S6. Associations of OH and frailty with dementia and death, considering supine hypertension in OH

	OH-free		With OH	
	**No. outcome**	**HR (95% CI)**	**No. outcome**	**HR (95% CI)**
**Dementia as the outcome**				
**Robust**	55	1.0 (reference)	19	2.01 (1.15-3.53)[a]
**Frail**	172	1.74 (1.28-2.38)[b]	50	2.68 (1.80-3.98)[b]
**Death without dementia as the outcome**				
**Robust**	187	1.0 (reference)	32	1.25 (0.87-1.78)
**Frail**	405	1.31 (1.09-1.57)[b]	96	1.63 (1.27-2.09)[b]
**Transition from dementia to death as the outcome**				
**Robust**	31	1.0 (reference)	16	1.43 (0.79-2.59)
**Frail**	124	1.27 (0.85-1.90)	34	0.90 (0.55-1.46)

The numbers of people without and with OH are 2136 and 386. The number of people missing OH status is 181.
Results are from multistate flexible parametric survival models with OH-free and robust people as the reference group. HRs (95% CIs) were adjusted for age, sex, education, smoking status, body mass index categories, hypertension, diabetes, atrial fibrillation, ischaemic heart disease, heart failure, and cerebrovascular disease.
When dementia was the outcome, HR of the interaction term between OH and frailty = 0.86 (0.47 to 1.58), P-value = 0.634. Measure of effect modification on additive scale (RERI) = -0.10 (-1.38 to 1.18), P-value = 0.878.
When death without dementia was the outcome, HR of the interaction term between OH and frailty = 1.15 (0.75 to 1.76), P-value = 0.514. Measure of effect modification on additive scale (RERI) = 0.07 (-0.49 to 0.62), P-value = 0.809.
When transition from dementia to death was the outcome, HR of the interaction term between OH and frailty = 0.47 (0.24 to 0.94), P-value = 0.033. Measure of effect modification on additive scale (RERI) = -0.81 (-1.83 to 0.21), P-value = 0.120.
[a]P-value <0.05.
[b]P-value <0.01.
CI = confidence interval; HR = hazard ratio; OH = orthostatic hypotension.

Table S7. Associations of OH and frailty with dementia and death, adjusted for categories of sitting blood pressure levels and use of antihypertensives

	OH-free		With OH	
	**No. outcome**	**HR (95% CI)**	**No. outcome**	**HR (95% CI)**
**Dementia as the outcome**				
**Robust**	41	1.0 (reference)	33	2.24 (1.39-3.61)[a]
**Frail**	153	1.97 (1.37-2.83)[a]	69	2.55 (1.71-3.79)[a]
**Death without dementia as the outcome**				
**Robust**	166	1.0 (reference)	53	1.11 (0.83-1.48)
**Frail**	357	1.28 (1.06-1.55)[b]	144	1.57 (1.26-1.96)[a]
**Transition from dementia to death as the outcome**				
**Robust**	24	1.0 (reference)	23	1.40 (0.79-2.47)
**Frail**	107	1.30 (0.85-2.01)	51	1.15 (0.72-1.84)

Results are from multistate flexible parametric survival models with OH-free and robust people as the reference group. HRs (95% CIs) were adjusted for age, sex, education, smoking status, body mass index categories, diabetes, atrial fibrillation, ischaemic heart disease, heart failure, cerebrovascular disease, and categories of sitting blood pressure levels and use of antihypertensives.

When dementia was the outcome, HR of the interaction term between OH and frailty = 0.58 (0.34 to 0.98), P-value = 0.041. Measure of effect modification on additive scale (RERI) = -0.68 (-1.93 to 0.57), P-value = 0.285.

When death without dementia was the outcome, HR of the interaction term between OH and frailty = 1.18 (0.82 to 1.70), P-value = 0.385. Measure of effect modification on additive scale (RERI) = 0.18 (-0.27 to 0.62), P-value = 0.431.

When transition from dementia to death was the outcome, HR of the interaction term between OH and frailty = 0.61 (0.31 to 1.19), P-value = 0.148. Measure of effect modification on additive scale (RERI) = -0.56 (-1.49 to 0.37), P-value = 0.238.

[a]P-value <0.01.

[b]P-value <0.05.

CI = confidence interval; HR = hazard ratio; OH = orthostatic hypotension.

Table S8. Associations of OH and frailty with dementia and death, additionally adjusted for the use of antidepressants

	OH-free		With OH	
	**No. outcome**	**HR (95% CI)**	**No. outcome**	**HR (95% CI)**
**Dementia as the outcome**				
**Robust**	41	1.0 (reference)	33	2.36 (1.50-3.72)[a]
**Frail**	153	1.96 (1.40-2.75)[a]	69	2.57 (1.74-3.80)[a]
**Death without dementia as the outcome**				
**Robust**	166	1.0 (reference)	53	1.14 (0.85-1.53)
**Frail**	357	1.28 (1.05-1.55)[b]	144	1.56 (1.22-2.00)[a]
**Transition from dementia to death as the outcome**				
**Robust**	24	1.0 (reference)	23	1.42 (0.80-2.54)
**Frail**	107	1.27 (0.82-1.95)	51	1.13 (0.71-1.81)

Results are from multistate flexible parametric survival models with OH-free and robust people as the reference group. HRs (95% CIs) were adjusted for age, sex, education, smoking status, body mass index categories, hypertension, diabetes, atrial fibrillation, ischaemic heart disease, heart failure, cerebrovascular disease, and the use of antidepressants.
When dementia was the outcome, HR of the interaction term between OH and frailty = 0.58 (0.34 to 0.99), P-value = 0.047. Measure of effect modification on additive scale (RERI) = -0.76 (-1.99 to 0.46), P-value = 0.220.
When death without dementia was the outcome, HR of the interaction term between OH and frailty = 1.18 (0.82 to 1.70), P-value = 0.360. Measure of effect modification on additive scale (RERI) = 0.14 (-0.29 to 0.58), P-value = 0.517.
When transition from dementia to death was the outcome, HR of the interaction term between OH and frailty = 0.59 (0.31 to 1.14), P-value = 0.117. Measure of effect modification on additive scale (RERI) = -0.57 (-1.55 to 0.41), P-value = 0.256.
[a]P-value <0.01.
[b]P-value <0.05.
CI = confidence interval; HR = hazard ratio; OH = orthostatic hypotension.

Table S9. Associations of OH and frailty with dementia and death, excluding dementia and death cases in the first three years of the follow-up period

	OH-free		With OH	
	**No. outcome**	**HR (95% CI)**	**No. outcome**	**HR (95% CI)**
**Dementia as the outcome**				
**Robust**	38	1.0 (reference)	31	2.44 (1.50-3.97)[a]
**Frail**	120	1.89 (1.30-2.77)[a]	48	2.52 (1.63-3.87)[a]
**Death without dementia as the outcome**				
**Robust**	147	1.0 (reference)	45	1.08 (0.79-1.49)
**Frail**	276	1.26 (1.03-1.55)[b]	99	1.55 (1.20-2.02)[a]

Results are from multistate flexible parametric survival models with OH-free and robust people as the reference group. HRs (95% CIs) were adjusted for age, sex, education, smoking status, body mass index categories, hypertension, diabetes, atrial fibrillation, ischaemic heart disease, heart failure, and cerebrovascular disease.

When dementia was the outcome, HR of the interaction term between OH and frailty = 0.53 (0.30 to 0.94), P-value = 0.029. Measure of effect modification on additive scale (RERI) = -0.83 (-2.26 to 0.60), P-value = 0.255.

When death without dementia was the outcome, HR of the interaction term between OH and frailty = 1.17 (0.79 to 1.74), P-value = 0.432. Measure of effect modification on additive scale (RERI) = 0.21 (-0.25 to 0.67), P-value = 0.380.

[a]P-value <0.01.

[b]P-value <0.05.

CI = confidence interval; HR = hazard ratio; OH = orthostatic hypotension.

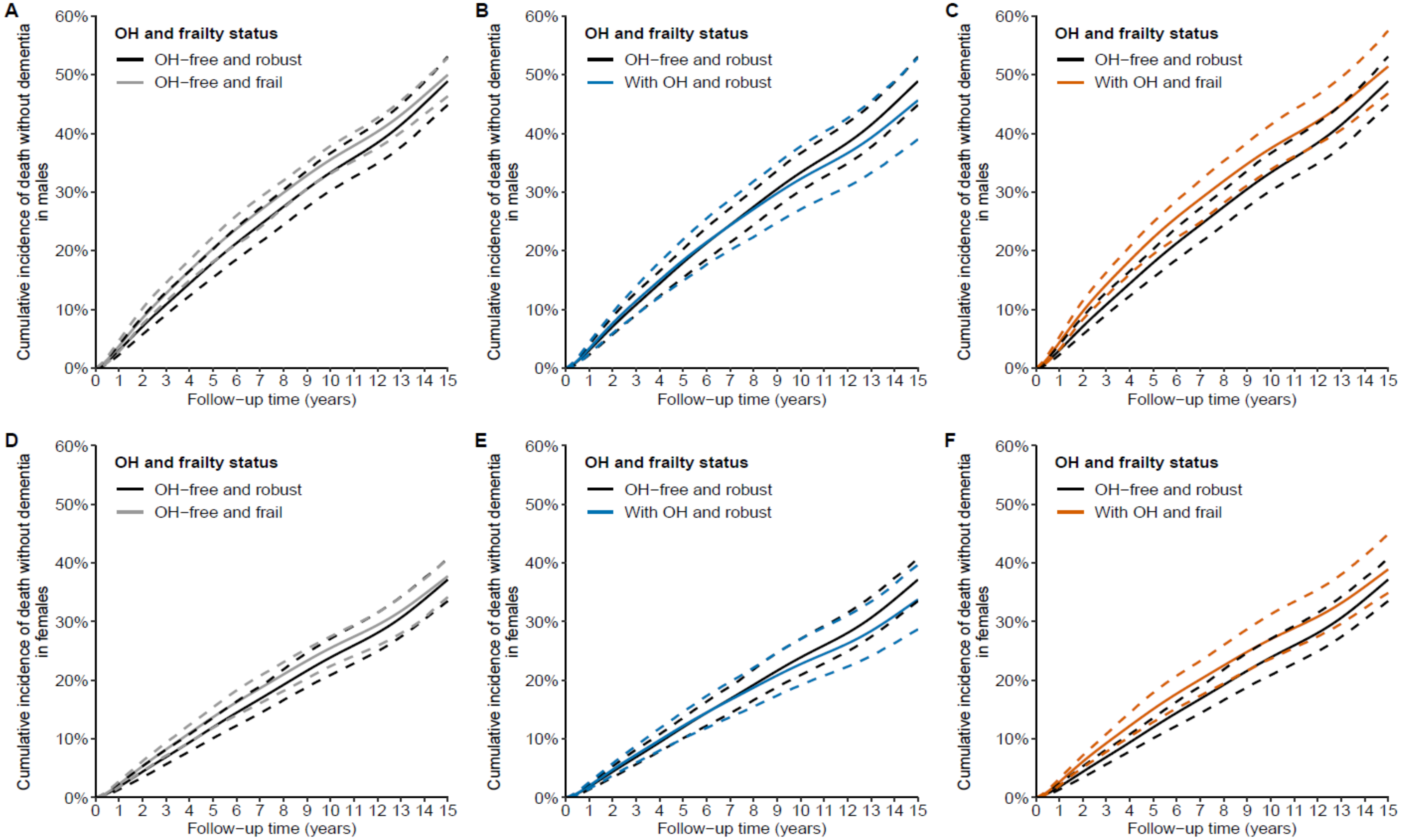

Figure S1. Standardised cumulative incidence of death without dementia by OH and frailty status. A-C, Standardised cumulative incidence of death without dementia in males; D-F, Standardised cumulative incidence of death without dementia in females. Dashed lines are 95% confidence intervals.

OH = orthostatic hypotension.

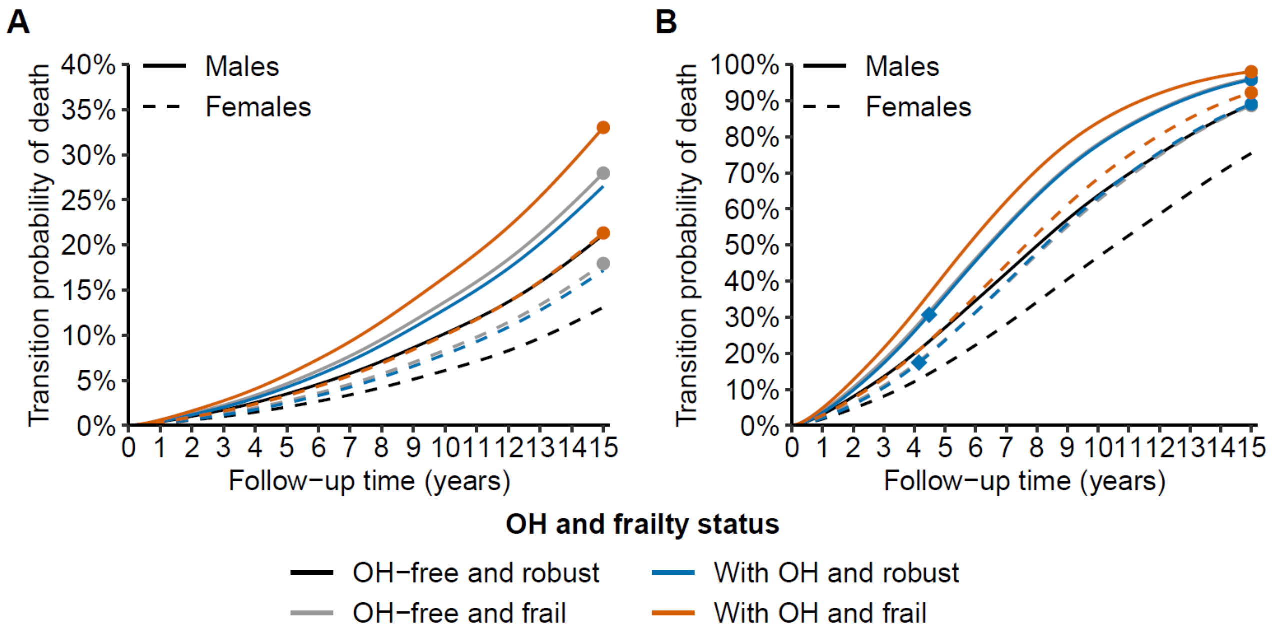

Figure S2. Transition probability of death in males and females by OH and frailty status. A, Transition probability of death for people aged 65 years; B, Transition probability of death for people aged 85 years.

Filled diamond-shaped symbols indicate when the differences in transition probabilities compared with the OH-free and robust group start being significant. Without this symbol, the differences are significant from the beginning of the follow-up time.

Solid circles indicate when the differences in transition probabilities compared with the OH-free and robust group stop being significant.

Predictions at age 65 years are made for people with a university educational level, not smoking currently, having normal weight, having hypertension, and without diabetes, atrial fibrillation, ischaemic heart disease, heart failure, and cerebrovascular disease. Predictions at age 85 years are made for people with the same characteristics except for the educational level being high school level. The values of the covariates were chosen based on their mean or most frequent values.

OH = orthostatic hypotension.
